# Comparative Transcriptomics Reveals Jasmonic Acid-Associated Metabolism Related to Cotton Fiber Initiation

**DOI:** 10.1371/journal.pone.0129854

**Published:** 2015-06-16

**Authors:** Liman Wang, Youmin Zhu, Wenjing Hu, Xueying Zhang, Caiping Cai, Wangzhen Guo

**Affiliations:** State Key Laboratory of Crop Genetics & Germplasm Enhancement, Hybrid Cotton R & D Engineering Research Center, MOE, Nanjing Agricultural University, Nanjing, Jiangsu, China; National Taiwan University, TAIWAN

## Abstract

Analysis of mutants and gene expression patterns provides a powerful approach for investigating genes involved in key stages of plant fiber development. In this study, lintless-fuzzless XinWX and linted-fuzzless XinFLM with a single genetic locus difference for lint were used to identify differentially expressed genes. Scanning electron microscopy showed fiber initiation in XinFLM at 0 days post anthesis (DPA). Fiber transcriptional profiling of the lines at three initiation developmental stages (-1, 0, 1 DPA) was performed using an oligonucleotide microarray. Loop comparisons of the differentially expressed genes within and between the lines was carried out, and functional classification and enrichment analysis showed that gene expression patterns during fiber initiation were heavily associated with hormone metabolism, transcription factor regulation, lipid transport, and asparagine biosynthetic processes, as previously reported. Further, four members of the allene-oxide cyclase (AOC) family that function in jasmonate biosynthesis were parallel up-regulation in fiber initiation, especially at -1 DPA, compared to other tissues and organs in linted-fuzzed TM-1. Real time-quantitative PCR (RT-qPCR) analysis in different fiber mutant lines revealed that *AOCs* were up-regulated higher at -1 DPA in lintless-fuzzless than that in linted-fuzzless and linted-fuzzed materials, and transcription of the *AOCs* was increased under jasmonic acid (JA) treatment. Expression analysis of JA biosynthesis-associated genes between XinWX and XinFLM showed that they were up-regulated during fiber initiation in the fuzzless-lintless mutant. Taken together, jasmonic acid-associated metabolism was related to cotton fiber initiation. Parallel up-regulation of *AOCs* expression may be important for normal fiber initiation development, while overproduction of *AOCs* might disrupt normal fiber development.

## Introduction

*Gossypium hirsutum* (cotton) is an important economic crop and is a major source of both natural textile fiber and cottonseed oil. Cotton fibers are single-celled trichomes from individual epidermal cells on the outer integument of the ovules. Fiber development involves four distinct but overlapping stages: initiation, elongation/primary cell wall (PCW) synthesis, secondary cell wall (SCW) deposition and maturation. Fiber cell initiation usually begins around anthesis and is characterized by the enlargement and protrusion of epidermal cells from the ovular surface. All epidermal cells have the potential to become fibers, but only about 30% will differentiate into commercially viable lint fibers that can be spun [1,2]. During 5–25 days post anthesis (DPA), fiber cells elongate rapidly with peak growth rates of over 2 mm per day [3,4]. During secondary cell wall deposition (20–45 DPA), cellulose biosynthesis predominates until fibers contain approximately 90% cellulose and the cell wall is adequately thickened. During the final maturation stage (45–50 DPA) fibers quickly dehydrate to become mature [1,2,5].

Fiber initiation progresses through two morphological phases: (1) differentiation of the pre-fiber from the epidermal cell ovule, and (2) expansion and protuberance of the pre-fiber cell [6]. The second stage involves numerous genes that control the cell cycle, hormone regulation, cytoskeletal features, signal transduction, and formation and deposition of complex carbohydrates and cell wall proteins [7]. In addition, maintaining homeostasis of reactive oxygen species (ROS) is crucial for cotton fiber initiation [8].

Mutant organisms are powerful tools for elucidating the molecular mechanisms of important genes. In cotton, important fiber mutants have been characterized including fuzzless-lintless (*fl*), such as MD17 [9], SL 1-7-1 [10], Xz-142 WX [11], naked seed (*N1* and *n2*) [12,13], ligon-lintless (*Li1* and *Li2*) [14,15], and immature fiber (*im*) [16]. A combination of fiber development mutants and high-throughout gene expression analysis has identified genes involved in key stages of fiber development. Padmalatha et al. [17] compared transcriptome analysis of *fl* with wild-type *G*. *hirsutum* cv. MCU5 during fiber initiation (0 DPA) and found that genes involved in calcium and phytohormone-mediated signalling pathways played crucial regulatory roles during fiber differentiation, while mitochondria mediate energy metabolism during fiber elongation. Shi et al. [18] found ethylene biosynthesis to be one of the major biochemical pathways to be upregulated during fiber elongation by combining cDNA library sequencing and microarray analysis. Transcription profiling of the *im* mutant and its near-isogenic line (NIL) TM-1 during fiber secondary cell wall development led to the identification of an asynchronous fiber development at the transition between secondary cell wall biosynthesis and earlier stages [19].

XinWX is a lintless-fuzzless fiber mutant, showing the similar phenotype with Xz-142 WX. XinFLM is a fuzzless-linted mutant isolated from XinWX. Genetic analysis revealed that XinFLM was a dominant mutant that only differed from XinWX at a single locus for lint [20]. The two mutant lines showed no significant phenotypic differences except for the mature bolls (S1 Fig). Seeds of XinFLM have lint but no fuzz, while those of XinWX are lintless and fuzzless. In this study, we performed a comparative analysis of the transcriptional profile during fiber initiation development of lintless-fuzzless XinWX and linted-fuzzless XinFLM using 28K cDNA microarrays. Differentially expressed genes (DEGs) were identified and their potential functional roles during fiber differentiation and initiation were investigated. Besides largerly agreed with previously reported, this study established a foundation for elucidating the functional role of allene oxide cyclases (AOCs) in the regulation of the development of cotton fiber initation, and suggested that jasmonic acid-associated metabolism was invovled in cotton fiber initiation.

## Materials and Methods

### Plant materials

Plant materials used in this study contain Xinxiangxiaoji lintless-fuzzless and linted-fuzzless isogenic lines (XinWX and XinFLM, respectively); Upland cotton genetic standard line Texas Marker-1(*G*. *hirsutum* acc. TM-1); Xuzhou-142 (Xz-142) and Xuzhou-142 lintless-fuzzless mutant (Xz-142 WX); recessive naked seed n2 line. All plants were grown under standard field conditions at PaiLou experimental field, Nanjing Agricultural University, Jiangsu Province, China. All necessary permits for collecting XinFLM, Xz-142 and TM-1 were obtained from Nanjing Agricultural University, others from Cotton Research Institute, Chinese Academy of Agricultural Sciences, China. Flowers were hand-pollinated and tagged at 0 DPA, and cotton bolls were collected at -3, -1, 0, 1 and 3 DPA.

*G*. *hirsutum* acc. TM-1 was used for tissue/organ expression analysis. Roots, stems and leaves were collected from two-week-old seedlings, and ovules and fibers were excised from developing flower buds or bolls. *G*. *hirsutum* cv. Jinmian 19 was used for JA treatment, and seedlings at the four leaf stage were treated with 100 μM JA (with water as a control). Leaves were harvested at the appropriate times (0, 0.5, 1, 2, 4, 6, 8, 10, 12, and 24 h following treatment). All tissues collected were flash-frozen in liquid nitrogen and stored at –70°C.

### Sample preparation for scanning electron microscopy (SEM) analysis

Ovule samples from XinWX and XinFLM at -1, 0, and 1 DPA were dehydrated serially for 30 min in 30%, 50%, 70%, 80%, 90% and 100% ethanol, then incubated in isoamyl acetate for 3 × 30 min. Samples were freeze-dried using an ES-2030 (Hitachi, Japan), sputter-coated with silver using an E-1010/E-1020 ion sputter (Hitachi, Japan), and imaged using an S-3000N SEM (Hitachi, Japan) [21].

### RNA isolation

The CTAB-acidic phenolic method [22] was used to extract total RNA that was purified using the NucleoSpin RNA clean-up kit (MACHEREY-NAGEL, Germany). Spectrophotometry and 1.2% agarose formaldehyde denaturing gel electrophoresis were used to monitor RNA quantity and integrity.

### RNA amplification and labeling

The cRNA amplification and labeling Kit (CapitalBio, China) was used to amplify and label RNA following the manufacturer’s protocol. cDNA samples from XinWX and XinFLM collected during the same fiber developmental stage were labeled with Cy3 and Cy5-dCTP, respectively, and these dyes were interchanged to label cDNA samples in a dye swap experiment.

### Microarray hybridization and statistical analysis

Expression patterns were analyzed using the dual-dye 28 k cotton cDNA microarray platform (GPL8569) containing 29,184 probes from Upland cotton (*G*. *hirsutum* L. cv. Xuzhou 142) ovule and fiber samples collected between -3 and 25 DPA. Microarray hybridization was carried out as described [19] using -1, 0 and 1 DPA samples from XinWX and XinFLM. The experiment was finished by pairwise hybridizations between the two lines at the same sampling timepoint with two biogical replicates and a dye swap. All microarrays were scanned with a LuxScan 10 K/A scanner (CapitalBio) and array images were analyzed with LuxScan^TM^ 3.0 (CapitalBio). Microarray data have been deposited in the Gene Expression Omnibus of NCBI (GEO series accession number GSE59208; http://www.ncbi.nlm.nih.gov/geo/query/acc.cgi?acc=GSE59208).

The LIMMA program [23] based on the open source Bioconductor project (http://www.bioconductor.org) was used to perform statistical analyses. Feint spots were removed if the intensity was less than 400 units following background subtraction. The LOWESS [24] and Aquantile [25] methods were then used to normalize the data within and across the arrays, respectively. In order to analyze DEGs, normalized data were fitted to linear models in LIMMA and a moderated *t*-statistic (eBays) calculation was performed [26]. Hybridization signal ratios from each slide were used to determine DEGs between the two accessions at each time point, and single channel data *in silico* were compared to determine DEGs from the same accession at different time points. Genes were designated as differentially expressed if FDR <0.05 and the fold change ≥2. Blast2GO (http:// www.Blast2go.com/) was then used for functional categorization of DEGs, GO analysis, and enrichment analysis with Fisher’s exact test for each GO term, in which FDR *P* values (FDR <0.05) were used for the assessment of over-represented GO terms [27].

### Real time-quantitative PCR analysis

RNA samples were reverse transcribed into cDNA according to the manufacturer’s instructions. Reactions contained 2 μg RNA, 0.5 μg oligo (dT), 200 units M-MLV RT (Cat#M1705; Promega, USA), 1.25 μl dNTP (10 mM) and 25 units RNasin Ribonuclease Inhibitor (Cat# N2511; Promega, USA). Gene-specific real time-quantitative PCR (RT-qPCR) primers were designed and synthesized commercially (Genscript, China; S1 Table). The *His3* gene was used as a control and RT-qPCR was performed using a LightCycler FastStart DNA Master SYBR Green I kit (Roche, Switzerland) in an ABI7500 sequence detection system (Applied Biosystems, USA) according to the manufacturer’s protocol. The reaction system was as follows: 1μL cDNA; 0.5 μL forward or reverse primers; 10 μL 2 × SYBR; 8 μL ddH_2_O. The PCR program was as follows: initial denaturation at 95C for 10 min, then 40 cycles of 95C for 15 s, 58 C for 15 s, and 72C for 30 s. The expression of each gene relative to *His3* was calculated according to the formula: fold change = 2^-△Ct^ (△Ct = Ct _Target gene_—Ct _His3_) [28]. For all real-time PCR reactions, three technical replicates were performed in each of the three biological experiments.

### In vitro culture of cotton ovules

Bolls were collected from TM-1 at -1 DPA. The bolls were sterilized in 70% ethanol for 5~10 min and washed three times with sterile distilled water. Ovules were then removed from the bolls under sterile conditons and floated on 30mL liquid BT medium in the flask with 5μM IAA and 0.5μM GA_3_. For JA treatment, JA was added to the liquid BT medium at concentrations of 0.1, 0.5, and 2.5μM, respectively. The ovules were cultured in the dark at 30°C for 2 days. Three replicates were done in each assay.

## Results

### Fiber initiation differences between XinWX and XinFLM

**Fig 1:**
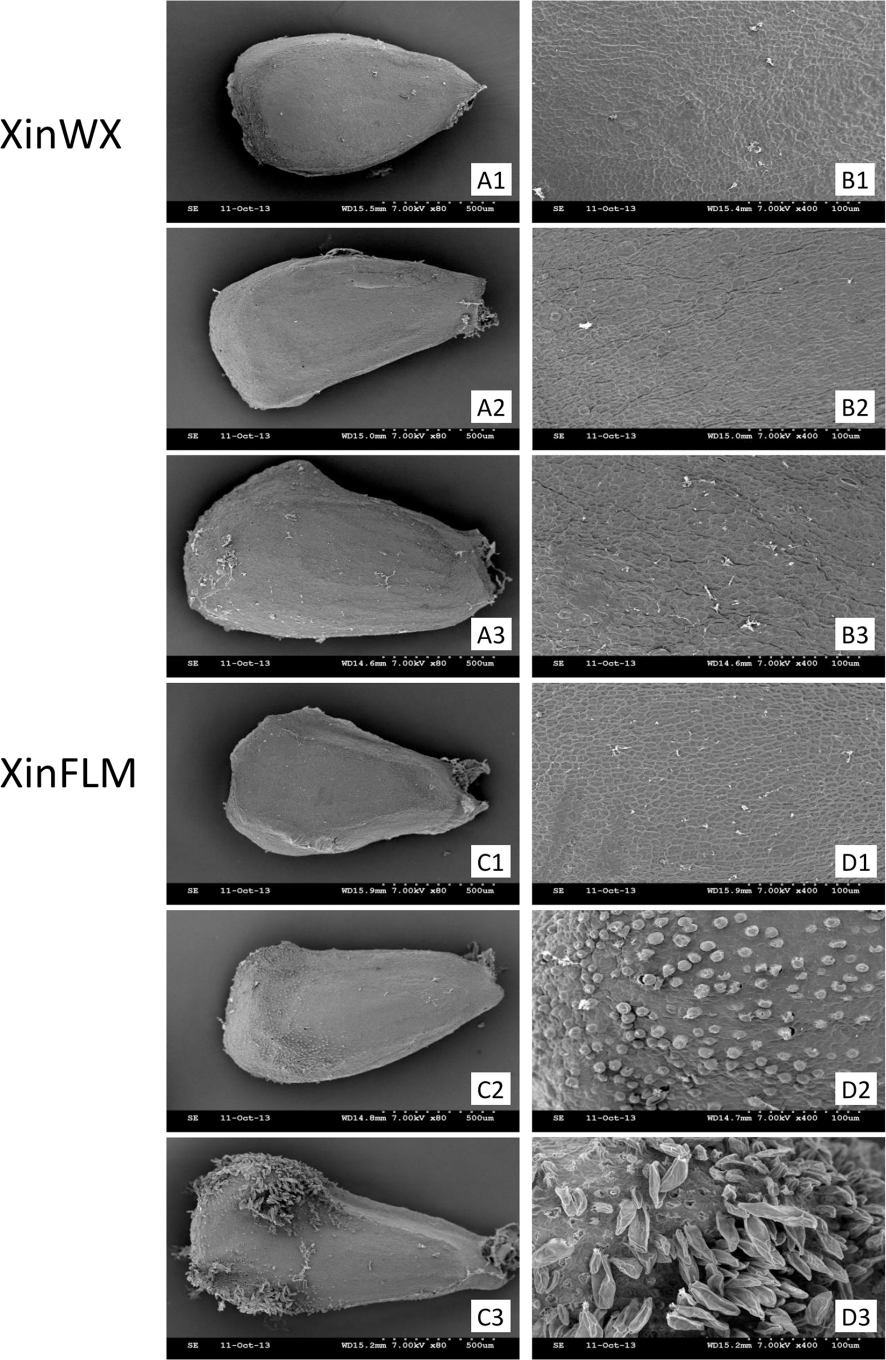
Scanning electron micrographs of ovules at -1, 0 and 1DPA in XinWX and XinFLM. (A, B) ovules of XinWX. (C, D) ovules of XinFLM. 1–3 indicate the day before anthesis (-1 DPA), the day of the anthesis (0 DPA), and the day after anthesis (1 DPA), respectively. Working distance (WD), accelerating voltage (7.00KV) and magnification (left: 50×; right: 400×) are indicated.

To identify differences between XinWX and XinFLM in fiber initiation, scanning electron microscopy (SEM) was used to compare the protrusion of epidermal cells during fiber initiation development (Fig 1). The entire ovule and flanking surface lacked any protrusion from -1 DPA to 1 DPA in XinWX (A1-A3, B1-B3). XinFLM also lacked spherical protrusions at the day before anthesis (C1, D1), but the whole ovule and flanking surface was covered in many spherical protrusions at 0 DPA (C2, D2) and 1 DPA (C3, D3), and elongated lint fibers were present at 1 DPA. This indicated that lintless-fuzzless XinWX failed to complete fiber initiation in the normal manner, and the fiber initiation phenotype of linted-fuzzless XinFLM was observed at the day of anthesis. Transcriptional differences between the two lines during fiber initiation (-1, 0, and 1 DPA) were subsequently investigated using a cDNA microarray approach.

### Differential gene expression in XinWX and XinFLM during fiber initiation

**Fig 2:**
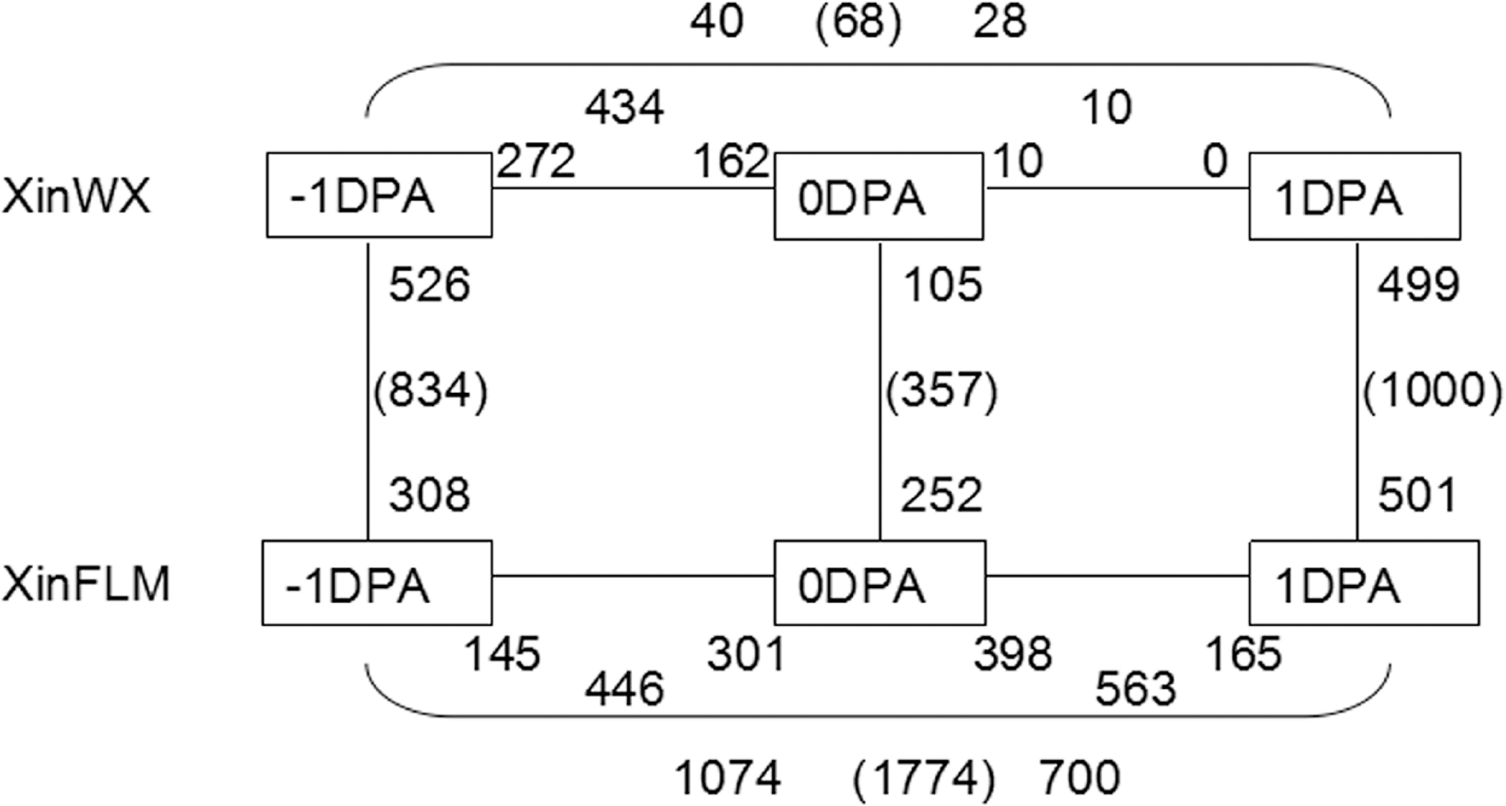
Number of differentially expressed genes within and between XinWX and XinFLM. Microarray analysis was performed on three replicates of each sample at three stages (represented by boxes) for both accessions. Numbers on or beside the line designate the number of upregulated genes (>2-fold, FDR <0.05) relative to the adjacent developmental stage or between two accessions. The total number of genes in each comparison is given in brackets.

Differences in gene expression during fiber initiation in the mutant lines were compared at -1, 0 and 1 DPA. Numbers of differentially expressed genes (DEGs) at adjacent time points within and between the lines were determined (Fig 2). The distribution of DEGs exhibiting a false discovery rate (FDR) <0.05 and a fold change 2 suggested the level of transcriptional variation differed significantly. In XinFLM, 1774 genes showed altered expression between 1 and -1 DPA, whereas only 68 DEGs were detected in XinWX, suggesting the gene expression profiles in XinWX had relatively few changes during the fiber initiation stage. Differences in gene expression between XinWX and XinFLM were most pronounced at -1 DPA (834 DEGs) and 1 DPA (1000 DEGs), while only 357 DEGs were detected at 0 DPA. In combination with the SEM results, these findings indicated that genes differentially expressed at 1 DPA may be involved in fiber elongation.

**Fig 3:**
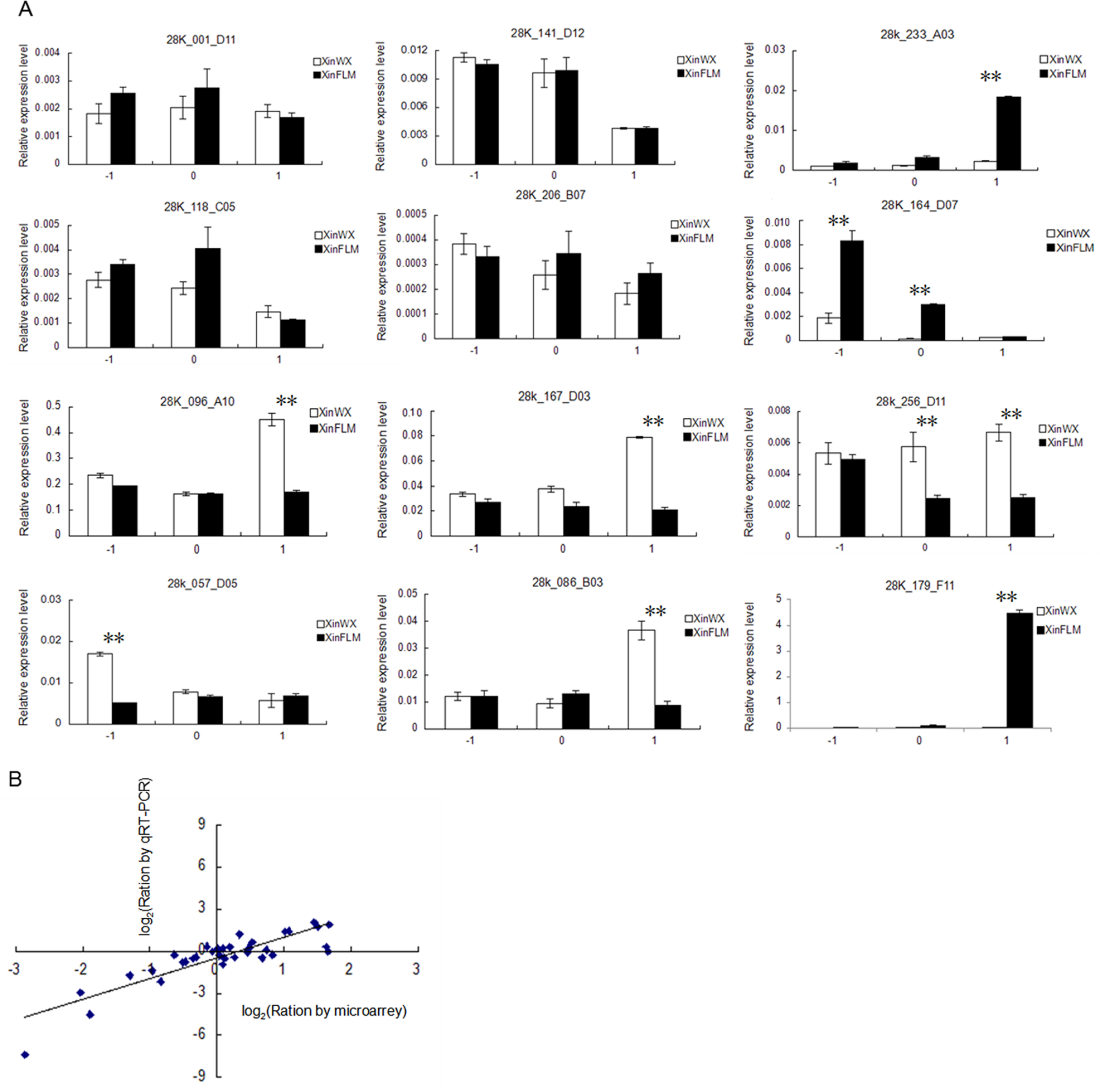
Comparison of gene expression ratios detected by microarray and RT-qPCR analyses. (A) Relative expression of 12 genes during fiber initiation. Mixtures of ovules and fibers at -1, 0, 1 DPA are shown. Error bars indicate ± SD of triplicate experiments. Three biological replicates were used for calculation. Significant differences at the 0.05 and 0.01 probability levels are represented by * and **, respectively. (B) Comparison of gene expression ratios from microarray and RT-qPCR analyses. Data from 12 genes at -1, 0, and 1 DPA from XinWX and XinFLM are shown. Log_2_ of the microarray expression ratios (x-axis) are plotted against log_2_ of the RT-qPCR expression ratios (y-axis).

Identified DEGs were further investigated using RT-qPCR. A total of 12 genes involved in various functional categories were selected to validate the microarray data. These included eight DEGs and four genes (28K_001_D11, 28K_118_C05, 28K_141_D12, 28K_206_B07) that did not exhibit differential expression between XinWX and XinFLM in microarray data analysis. For most genes, a similar expression pattern was obtained using both methods. Generally, the results of RT-qPCR were in agreement with the microarray data (correlation coefficient R = 0.87; Fig 3), which validated the microarray data.

### Gene ontology (GO) categories of differentially expressed genes within XinWX and XinFLM

Blast2GO was used to obtain GO functional annotation of all identified DEGs, with all of the genes on the chip as a reference group and DEGs as a test group to perform Fisher’s exact test (FDR <0.05). To obtain child GO and tip GO terms, the ‘show only most specific terms’ option was chosen. The main enriched GO terms from adjacent time points in both XinWX and XinFLM mutants are listed in Table 1 and discussed below. Complete information on over-represented GO terms is presented in S2 Table.

Between -1 and 0 DPA, XinWX and XinFLM shared some over-represented GO terms in common for up-regulated DEGs, for example ‘flavonoid biosynthesis process’ (GO:0009813, FDR = 0.00752 and 2.30E-16, respectively) at 0 DPA, which indicated that the two lines underwent some similar biological processes during this stage. During different intervals within the two lines, no over-represented GO terms were identified between 0 and 1 DPA or 1 and -1 DPA in XinWX, and between 1 and 0 DPA in XinFLM, for genes up-regulated at 1 DPA.

**Table 1:** Catalog of specific GO terms statistically over-represented during fiber initiation stages of XinWX and XinFLM.

Developmental stage comparison	Upregulated in XinWX or XinFLM	GO annotation	Functional categories	FDR
**0vs. -1dpa up in 0dpa**	**XinFLM**	GO:0009813	flavonoid biosynthetic process	2.30E-16
		GO:0005985	sucrose metabolic process	2.70E-04
		GO:0060416	response to growth hormone stimulus	0.00536
		GO:0008152	metabolic process	0.0064
		GO:0055114	oxidation-reduction process	1.03E-04
		GO:0005975	carbohydrate metabolic process	6.74E-07
		GO:0051301	cell division	0.0096
		GO:0019310	inositol catabolic process	0.04821
	**XinWX**	GO:0000280	nuclear division	0.00364
		GO:0009813	flavonoid biosynthetic process	0.00752
		GO:0009698	phenylpropanoid metabolic process	0.00364
**0vs. -1dpa up in -1dpa**	**XinFLM**	GO:0006529	asparagine biosynthetic process	1.34E-04
		GO:0009743	response to carbohydrate stimulus	2.54E-04
		GO:0042221	response to chemical stimulus	0.00135
		GO:0010200	response to chitin	0.00735
		GO:0009725	response to hormone stimulus	0.03591
	**XinWX**	GO:0010200	response to chitin	5.58E-09
		GO:0042221	response to chemical stimulus	5.00E-05
		GO:0000169	activation of MAPK activity involved in osmosensory signaling pathway	8.34E-04
		GO:0009867	jasmonic acid mediated signaling pathway	0.00328
		GO:0009863	salicylic acid mediated signaling pathway	0.00468
		GO:0009738	abscisic acid mediated signaling pathway	0.00485
**1vs.0dpa up in 0dpa**	**XinFLM**	GO:0042221	response to chemical stimulus	0.01595
	**XinWX**	NA	NA	NA
**1vs.0dpa up in 1dpa**	**XinFLM**	NA	NA	NA
	**XinWX**	NA	NA	NA
**1vs.-1dpa up in 1dpa**	**XinFLM**	GO:0055114	oxidation-reduction process	2.39E-05
		GO:0009813	flavonoid biosynthetic process	5.79E-05
		GO:0005975	carbohydrate metabolic process	1.70E-04
		GO:0005985	sucrose metabolic process	0.0096
		GO:0006869	lipid transport	0.02459
		GO:0043434	response to peptide hormone stimulus	0.04961
	**XinWX**	NA	NA	NA
**1vs. -1dpa up in -1dpa**	**XinFLM**	GO:0010200	response to chitin	8.23E-10
		GO:0009725	response to hormone stimulus	0.004169
		GO:0009755	hormone-mediated signaling pathway	0.008339
		GO:0046423	allene-oxide cyclase activity	0.005497
		GO:0009743	response to carbohydrate stimulus	2.01E-09
	**XinWX**	NA	NA	NA

NA, no result

FDR, false discovery rate.

In XinFLM, between -1 and 0 DPA, and -1 and 1 DPA, a large proportion of the DEGs that were up-regulated at 0 and 1 DPA involved in “sucrose metabolic processes” (GO:0005985, FDR = 2.7E-04 and 0.00960), “oxidation-reduction processes” (GO:0055114, FDR = 1.03E-04 and 2.39E-05) and “carbohydrate metabolic processes” (GO:0005975, FDR = 6.74E-07 and 1.70E-04) categories. This suggested that these processes were involved in fiber initiation. These GO terms included genes associated with fiber early development that were reported previously, such as sucrose synthase (Sus), which plays a rate-limiting role in the initiation of single-celled fibers [29]. Additionally, most genes that were differentially expressed during different intervals in XinFLM were associated with hormone metabolism. Between -1 and 0 DPA, many genes up-regulated at 0 DPA belonged to the category “response to growth hormone stimulus” (GO:0060416, FDR = 0.00536), likewise for those up-regulated at -1 DPA involved in “response to hormone stimulus” (GO:0009725, FDR = 0.03591). Between 1 and -1 DPA, many genes up-regulated at 1 DPA belonged to the category “response to peptide hormone stimulus” (GO:0043434, FDR = 0.04961), while many of those up-regulated at -1 DPA occupied the categories “response to hormone stimulus” (GO:0009725, FDR = 0.004169), “hormone-medicated signaling pathway” (GO:0009755, FDR = 0.008339), and "allene-oxide cyclase activity” (GO:0046423, FDR = 0.005497). These results indicated that plant hormones play an important role in fiber initiation development.

Between -1 and 0 DPA, over-represented GO terms for up-regulated DEGs at -1 DPA in XinWX included “jasmonic acid mediated signaling pathway” (GO:0009867, FDR = 0.00328), “salicylic acid mediated signaling pathway” (GO:0009863, FDR = 0.00468), and “abscisic acid mediated signaling pathway” (GO:0.0009738, FDR = 0.00485). These pathways may therefore negatively regulate fiber initiation. Abscisic acid (ABA) is known to have an inhibitory effect on fiber development [30, 31], however less information is available regarding the potential roles of jasmonic acid and salicylic acid in fiber development. Annotation of DEGs invovled in “jasmonic acid mediated signaling pathway” showed this GO term contained some genes such as bHLH transcription factor. In previous study, MYC2, a bHLH transcription factor, differentially modulates diverse jasmonate-dependent functions in *Arabidopsis* [32]. Here, the transcription factors enriched in the GO term of “jasmonic acid mediated signaling pathway” might be related to cotton fiber intiation development.

### Over-represented GO categories of differentially expressed genes between XinWX and XinFLM during fiber initiation

Functional annotation and enrichment analysis of DEGs between XinWX and XinFLM at -1, 0 and 1 DPA were performed using Blast2GO. The most specific GO terms (Table 2) are discussed below, and complete information on over-represented GO terms is presented in S3 Table.

**Table 2:** Specific GO terms enriched between XinFLM and XinWX at three time points.

Developmental stage		GO annotation	Functional Categories	FDR
**-1 DPA**	**up in XinFLM**	GO:0006529	asparagine biosynthetic process	8.56E-10
		GO:0009743	response to carbohydrate stimulus	4.97E-04
		GO:0009646	response to absence of light	1.20E-04
		GO:0016762	xyloglucan:xyloglucosyl transferase activity	4.07E-08
		GO:0006541	glutamine metabolic process	0.011596
	**up in XinWX**	GO:0055114	oxidation-reduction process	2.27E-11
		GO:0006006	glucose metabolic process	2.04E-11
		GO:0006732	coenzyme metabolic process	0.00683
		GO:0055085	transmembrane transport	0.0486
		GO:0016052	carbohydrate catabolic process	3.13E-10
		GO:0046506	sulfolipid biosynthetic process	0.00138
**0 DPA**	**up in XinFLM**	GO:0010200	response to chitin	0.000103
		GO:0006869	lipid transport	2.44E-04
		GO:0010876	lipid localization	2.44E-04
		GO:0071702	organic substance transport	0.00701
		GO:0009743	response to carbohydrate stimulus	0.0255
		GO:0009700	indole phytoalexin biosynthetic process	0.04749
	**up in XinWX**	GO:0009765	photosynthesis, light harvesting	0.02905
		GO:0060862	negative regulation of floral organ abscission	0.03751
		GO:0019310	inositol catabolic process	0.02905
		GO:0018298	protein-chromophore linkage	0.01193
**1 DPA**	**up in XinFLM**	GO:0006869	lipid transport	0.00000024
		GO:0010876	lipid localization	2.40E-07
		GO:0071702	organic substance transport	0.00389
		GO:0050625	2-hydroxy-1,4-benzoquinone reductase activity	0.004528246
		GO:0042335	tcuticle development	0.04312
	**up in XinWX**	GO:0009642	response to light intensity	9.30E-05
		GO:0046423	allene-oxide cyclase activity	2.12E-04
		GO:0006529	asparagine biosynthetic process	0.00237
		GO:0009939	positive regulation of gibberellic acid mediated signaling pathway	0.00818
		GO:0055085	transmembrane transport	0.01671

NA, no result

FDR, false discovery rate.

A comparison of DEGs between the two lines showed that the “response to carbohydrate stimulus” category was over-represented in XinFLM both at -1 and 0 DPA (GO:0009743, FDR = 4.97E-04 and 0.02550, respectively), while the “lipid transport” (GO:0006869, FDR = 2.44E-04, 2.40E-07) and “organic substance transport” (GO:0071702, FDR = 0.00710 and 0.00389) categories were simultaneously enriched at 0 and 1 DPA in this mutant line. These GO terms included lipid transfer proteins (LTPs) and transcription factors such as ERFs, WRKYs and Zinc-finger proteins which were indicative of a positive regulatory effect on early fiber development. At -1 DPA, the “xyloglucan: xyloglucosyl transferase activity” (GO:0016762, FDR = 4.07E-08) category was highly represented in XinFLM. Xyloglucan is known to be involved in cell wall loosening and expansion during fiber elongation [33]. At 1 DPA, the GO term “2-hydroxy-1,4-benzoquinone reductase activity” (GO: 0050625, FDR = 0.004528) was over-represented, which included the cotton benzoquinone reductase known to be up-regulated in cotton ovules during fiber initiation [34]. XinWX also generated some particularly over-represented GO categories, including “oxidation-reduction processes” (GO:0055114, FDR = 2.27E-11), “carbohydrate catabolic processes” (GO: 0016052, FDR = 3.13E-10) at -1 DPA, “response to stress” (GO: 0006950, FDR = 1.43E-06 and 2.12E-04) at -1 and 1 DPA, respectively, and “glutamate synthase (NADH) activity” (GO: 0016040, FDR = 0.004267 and 9.84E-05) at -1 and 1 DPA, respectively. During the early stages of fiber development, carbohydrate and energy metabolism categories were over-represented more than once. This was consistent with previous reports suggesting these processes provide the carbon skeletons (polysaccharides and fatty acids) needed for cell wall synthesis and are therefore important for fiber development [35,36]. At 1 DPA, the “allene-oxide cyclase activity” (GO:0046423, FDR = 2.12E-04) was enriched in XinWX, which was also enriched in XinFLM at 1 DPA compared to -1 DPA (GO:0046423, FDR = 0.005497). AOC is a key enzyme in the biosynthesis of JA [37]. Plant hormones such as indole-3-acetic acid (IAA), gibberellic acid (GA3) and brassinosteroid (BR) are known to play important roles in cotton fiber initiation [38,39], and these results suggest that JA may also be involved in the progression of fiber initiation.

### Expression of AOCs plays a central role in cotton fiber initiation

**Fig 4:**
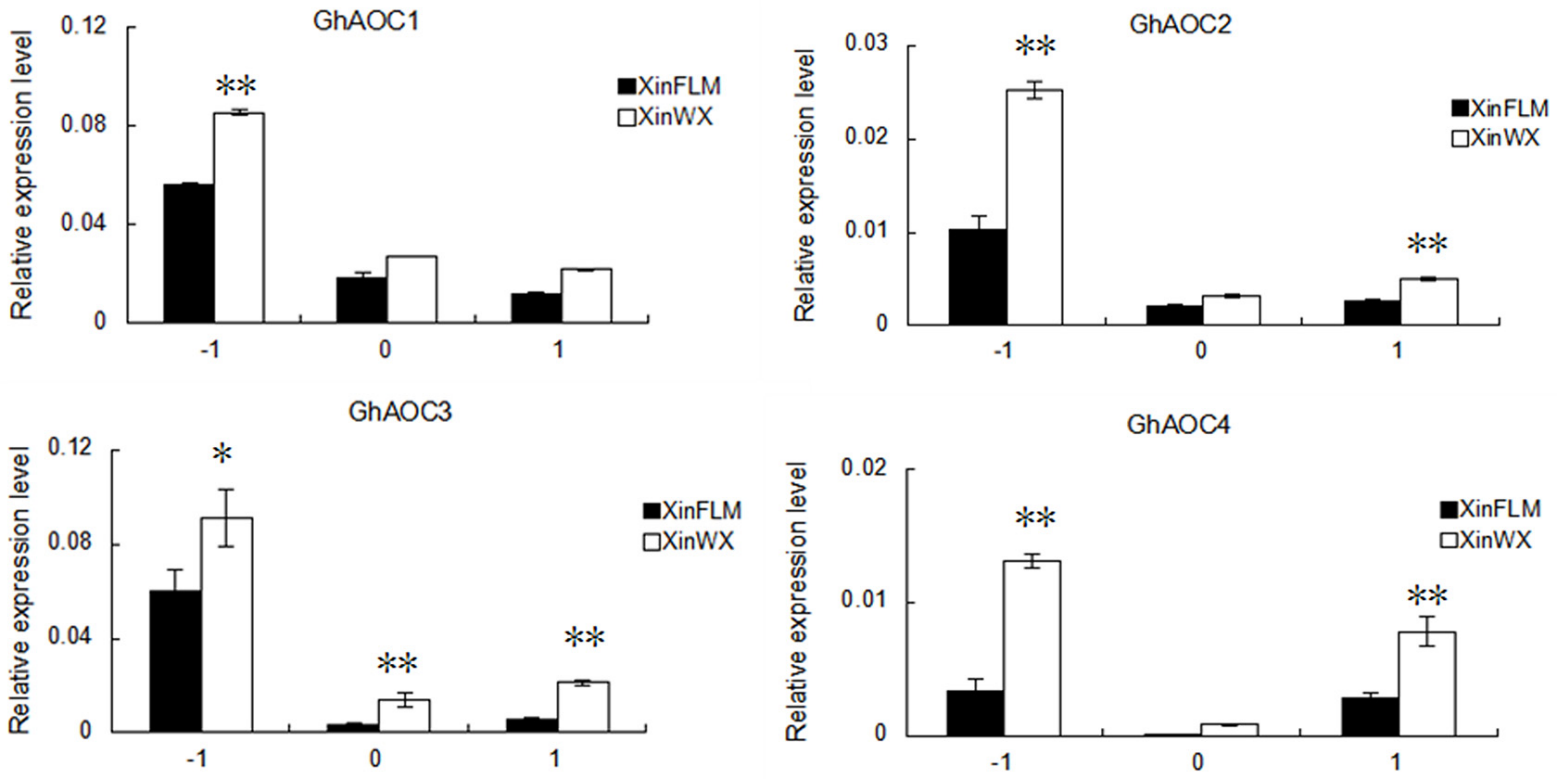
Comparison of *AOCs* expression in XinWX and XinFLM. Ovules at -1, 0 and 1 DPA are shown. Significant differences at the 0.05 and 0.01 probability levels are represented by * and **, respectively. Error bars indicate ± SD. The expression level was derived from triplicate experiments. Three biological replicates were used for calculation.

**Fig 5:**
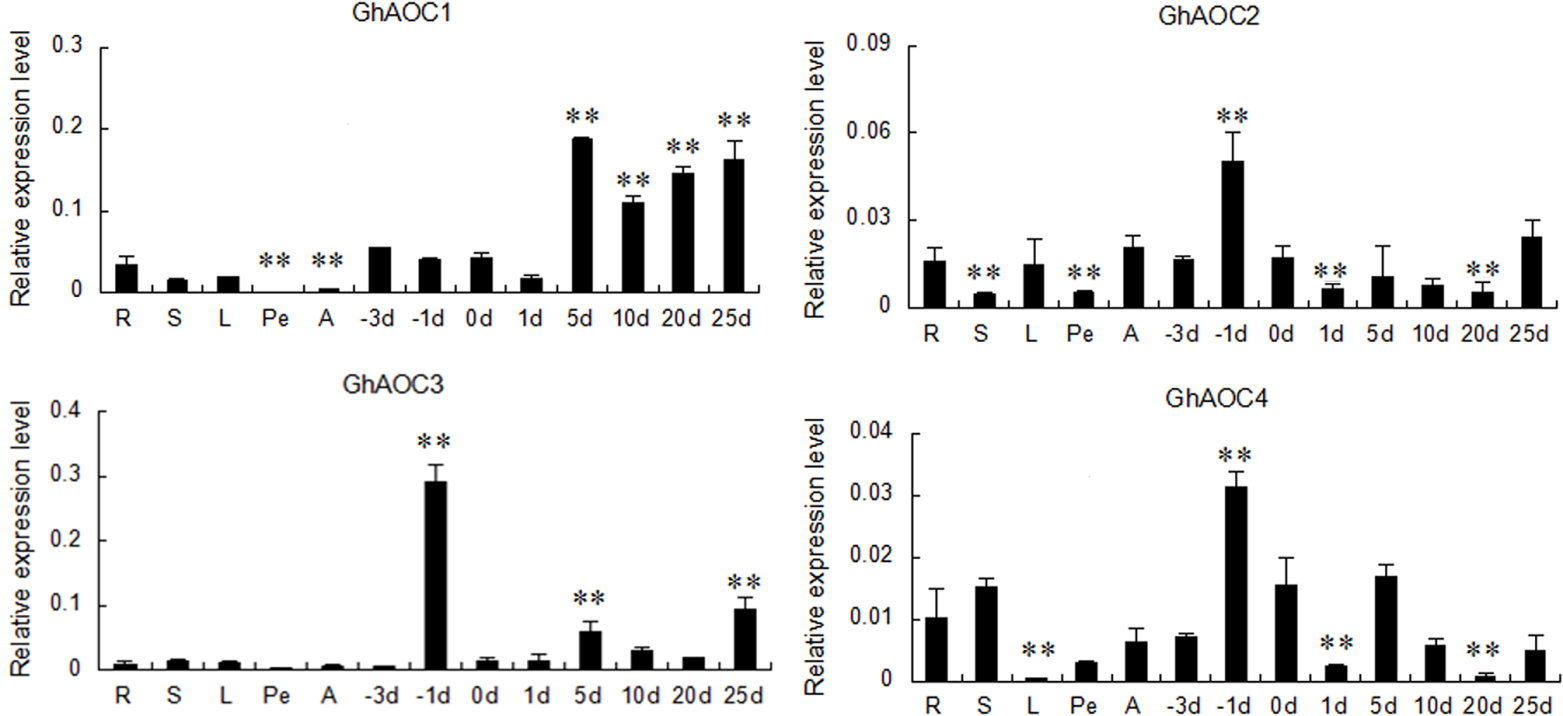
*AOCs* expression in different tissues and organs in TM-1. R, S, L, Pe, A, -3d, -1d, 0d, 1d, 5d, 10d, 20d, and 25d indicate root, stem, leaf, petal, anther, -3, -1, 0, 1 and 3 DPA ovules and 5, 10, 20 and 25 DPA fibers, respectively. Significant differences compared with the expression in root tissue at the 0.05 and 0.01 probability levels are represented by * and **, respectively. Error bars indicate ± SD of triplicate experiments. Three biological replicates were used for calculation.

The enriched allene-oxide cyclase activity GO category included four cotton ESTs, which corresponded to the four members (Gorai.004G046100, Gorai.004G046300, Gorai.004G046400, Gorai.008G185600) in *G*. *raimondii* (http://www.phytozome.net). In order to design the gene-specific primers, we downloaded the four AOC family genes from *G*. *raimondii*. Blast analysis showed that the *G*. *hirsutum* acc. TM-1 orthlog of Gorai.004G046100 (*GhAOC*) has been cloned previously [40], and was named as *GhAOC1* in this study, the other three *AOCs* in TM-1 were named *GhAOC2*, *GhAOC3*, and *GhAOC4* (corresponding to Gorai.004G046300, Gorai.004G046400, and Gorai.008G185600, respectively). Using gene-specific primers, it was confirmed that all four *AOCs* were upregulated during fiber initiation in XinWX compared with XinFLM, especially at -1 DPA (Fig 4), and all four *AOCs* were preferentially expressed in fiber tissues (Fig 5). *GhAOC1* was predominantly expressed during fiber development, especially during fiber elongation and secondary cell wall deposition, while expression in root, stem, and leaf was moderate. The *GhAOC2* transcript was much more abundant during fiber initiation (-3 to 1 DPA, with peak expression at -1 DPA), and also in fibers, roots, leaves and anther tissues at 25 DPA. *GhAOC3* expression also peaked at -1 DPA, and expression was much lower at 5 and 25 DPA in fibers, while expression was negligible in other tissues and organs compared to the peak at -1 DPA. *GhAOC4* was preferentially expressed in roots, stems and fibers during the early stages of development (-3 to 5 DPA), with expression again peaking at -1 DPA. Taken together, all four *AOCs* were preferentially expressed during the fiber initiation stage, especially at -1 DPA.

**Fig 6:**
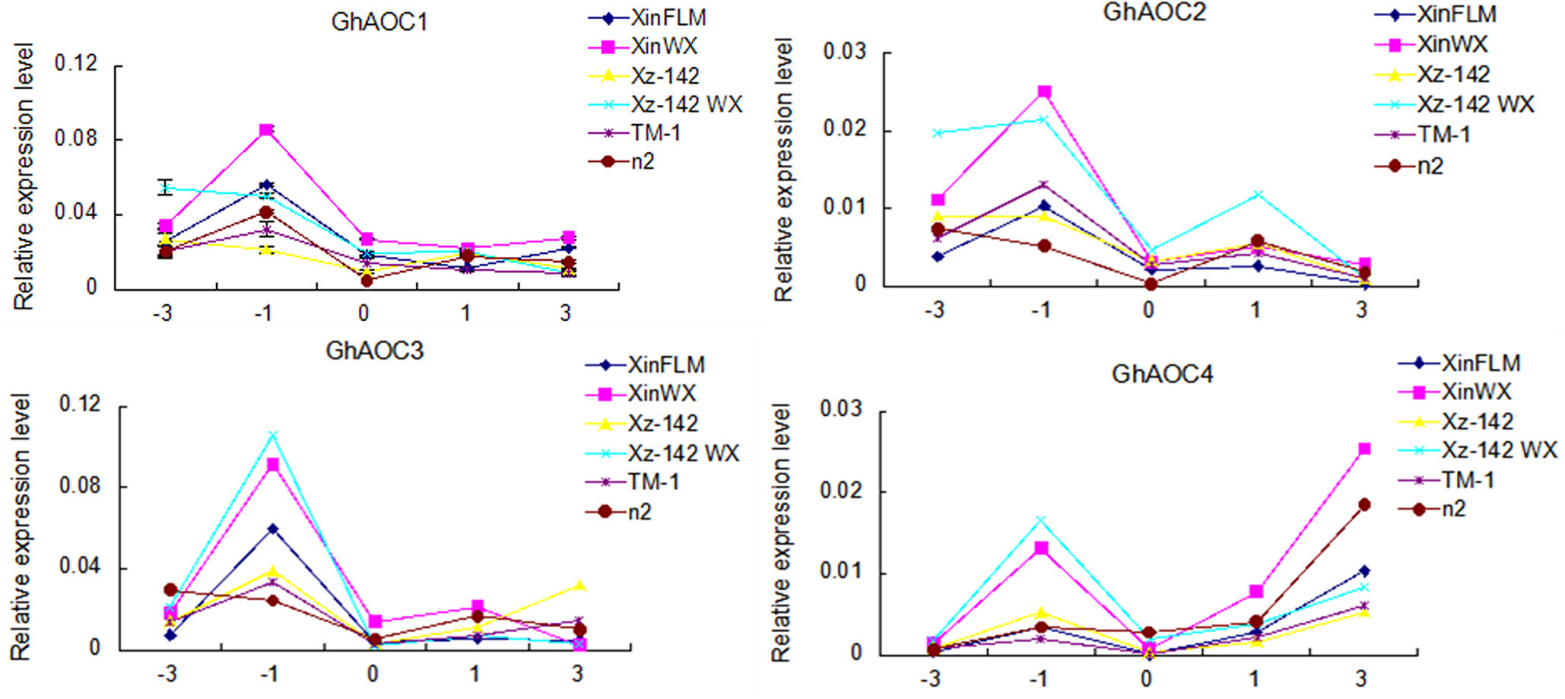
Relative expression of *AOCs* in fiber mutant lines during the early stages of fiber development. Mixtures of ovules and fibers at -3, -1, 0, 1 and 3 DPA are shown in x-axis. Error bars indicate ± SD of triplicate experiments. Three biological replicates were used for calculation.

To further investigate the role of *AOCs* in fiber initiation, their expression levels were measured between -3 and 3 DPA in different fiber mutant lines (Fig 6). Analysis of RT-qPCR data showed that *AOCs* exhibited a similar expression profile in all mutant lines tested. *GhAOC1*, *GhAOC2* and *GhAOC3* were preferentially expressed in ovules at -1 DPA, while *GhAOC4* mRNA was abundant at both -1 and 3 DPA. *GhAOC2*, *GhAOC3*, and *GhAOC4* were more highly expressed in lintless-fuzzless lines (XinWX, Xz-142 WX) (P<0.01) than in linted-fuzzless lines (XinFLM, n2) and linted-fuzzed lines (TM-1, Xz-142) at -1 DPA. In contrast, *GhAOC1* was more highly expressed in XinWX and Xz-142 WX than in NILs XinFLM and Xz-142, and expression was lowest in TM-1 and n2 (S4 Table). In summary, expression of *AOCs* in lintless-fuzzless mutants was generally higher than in linted-fuzzless and linted-fuzzed lines. These results, in combination with expression data from different tissues and organs in TM-1, suggested that upregulation of four *AOCs* was required for normal fiber initiation, but excessive overproduction of *AOCs* may lead to a suppressive effect on fiber initiation.

### Expression of AOCs is stimulated by JA

**Fig 7:**
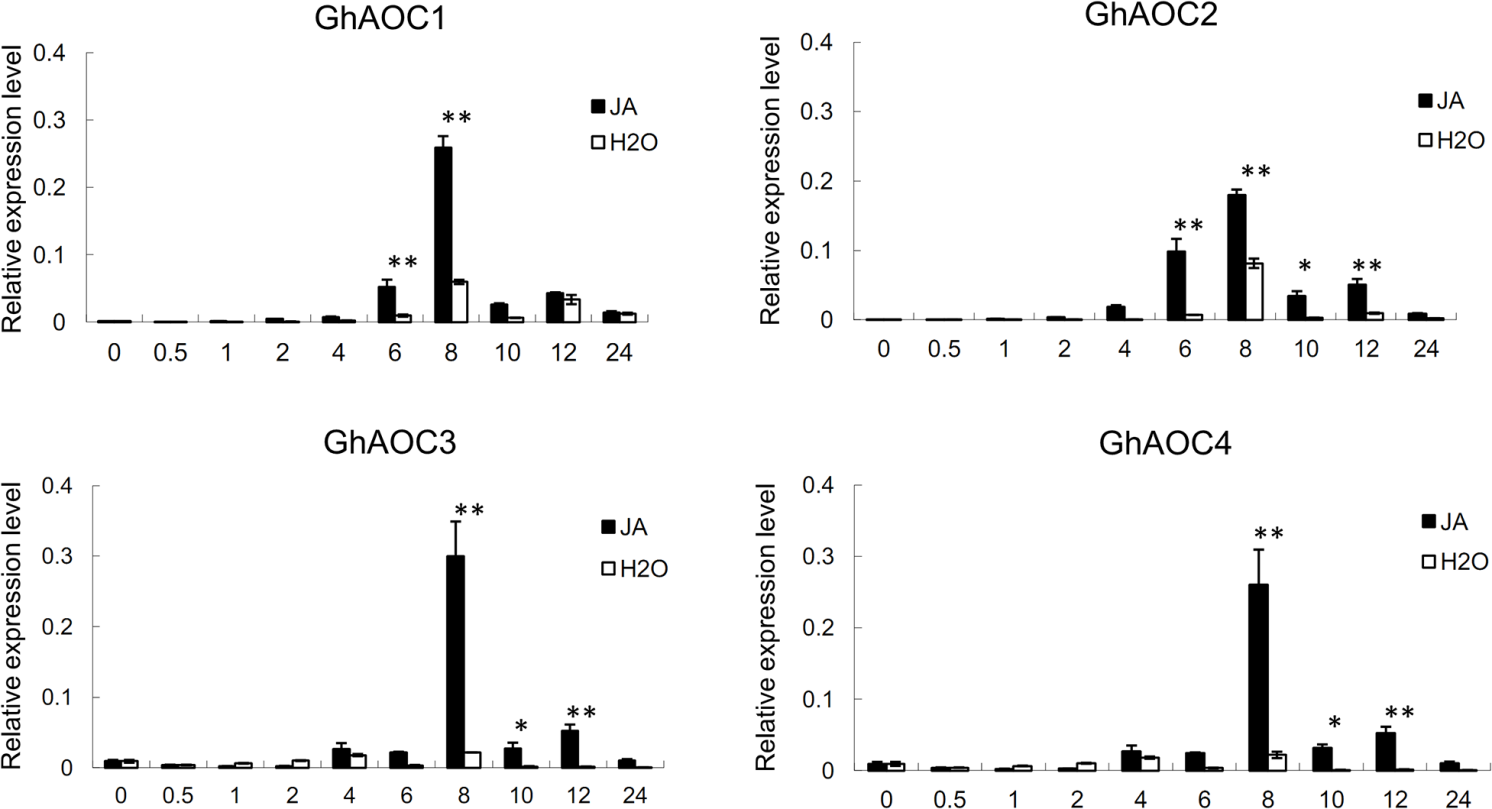
Expression of *AOCs* following JA treatment. The timepoints after initial JA treatment are shown, and treatment with water was used as a control. Significant differences at the 0.05 and 0.01 probability levels are represented by * and **, respectively. Error bars indicate ± SD of triplicate experiments. Three biological replicates were used for calculation.

In plants, AOCs are known to play a crucial role in JA biosynthesis. In order to investigate the relationship between expression of *AOCs* and JA, RT-qPCR was performed on leaf RNA of *G*. *hirsutum* cv. Jinmian 19, which was treated with JA for different time periods (with water as a control; Fig 7). All four *AOCs* exhibited a similar expression profile and all were significantly up-regulated following JA treatment, with a peak at 8 h after exposure.

### JA biosynthesis-associated genes were up-regulated during fiber initiation in the fuzzless-lintless mutant

**Fig 8:**
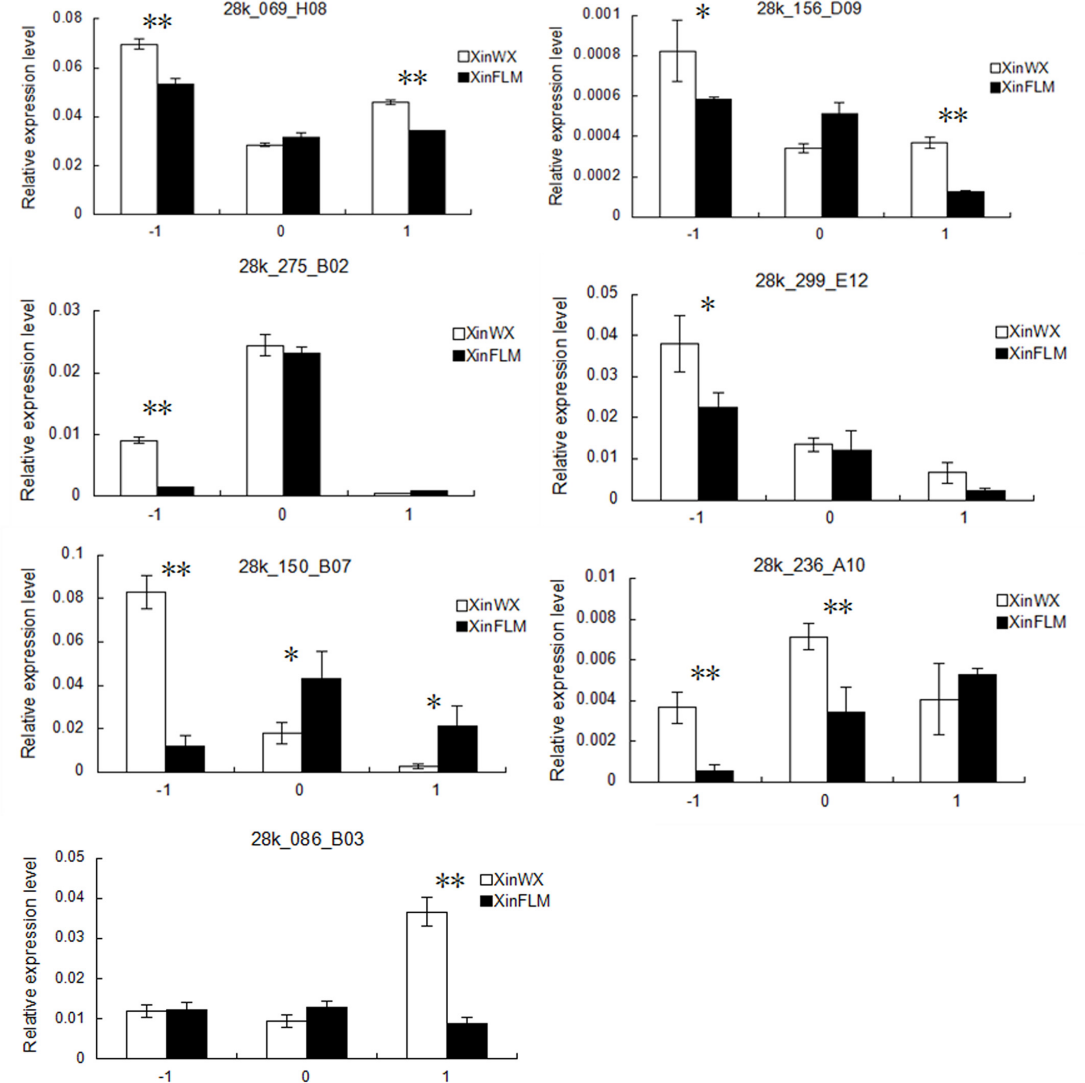
Differentially expressed genes involved in JA biosynthesis and signal transduction. Mixtures of ovules and fibers at -1, 0, and 1 DPA are shown in x-axis. Error bars indicate ± SD. Significant differences at the 0.05 and 0.01 probability levels are represented by * and **, respectively. The expression level was derived from triplicate experiments. Three biological replicates were used for calculation.

To determine whether JA biosynthesis was co-regulated during fiber initiation, seven genes (AOS, 28K_069_H08 and 28K_156_D09; LOX, 28K_275_B02 and 28K_150_B07; OPR, 28K_299_E12; GH3, 28K_086_B03 and 28K_236_A10), which were differentially expressed between XinWX and XinFLM in the microarrays and their homologs reported previously were involved in JA biosynthesis in other plants [41], were subjected to RT-qPCR analysis (Fig 8). Other than 28K_086_B03 which showed significant expression differences at 1 DPA, the other six DEGs showed significantly higher expression in XinWX than in XinFLM between -1 and 1 DPA, especially at -1 DPA. Of these, two ESTs (28K_086_B03 and 28K_236_A10), encoding GH3 proteins, are known to be important for jasmonate signaling [35, 36]. GH3 genes are induced by auxin and conjugate JA with amino acids. GH3.11 conjugates JA with isoleucine, and the resultant jasmonoyl-L-isoleucine (JA-Ile) is a crucial signalling molecule for the major jasmonate signaling pathway involving *COI1* [42]. GH3.3, GH3.5, and GH3.6 also conjugate JA to various amino acids and by doing so regulate JA homeostasis in *Arabidopsis* adventitious root initiation [43]. These results and the expression profiles of the *AOCs* suggested that the JA biosynthesis pathway is up-regulated during fiber initiation in the fuzzless-lintless mutant, who may result in overproduction of JA in XinWX compared to XinFLM.

## Discussion

### Several key genes and pathways are responsible for progression of fiber initiation

Cotton fiber initiation is a complex biological process involving genes controlling the cell cycle, hormone regulation, signal transduction, cytoskeletal features, and formation and deposition of complex carbohydrates and cell wall proteins [7]. Transcription factors are particularly important for fiber cell initiation [44]. *GhMYB25*, *GhMYB25-like*, and *GhHD1* were previously identified as regulators of lint fiber initiation [45], and silencing of *GhMYB25* in cotton led to delayed fiber initiation and formation of shorter fibers [46], and overexpression of *GhHD1* stimulated fiber initiation, while silencing delayed this process [47]. In addition to transcription factors, phytohormones play key roles in plant development and have been well-studied. At anthesis, ovules initiate fiber development in cultures supplemented with both IAA and GA3, whereas ABA, ethylene, and cytokinins inhibit fiber development [30,31,35]. Numerous genes involved in IAA, GA, and BR biosynthesis and signalling pathways have been cloned and confirmed to be associated with the development of fiber initiation [48,49]. Additionally, genes involved in calcium signalling pathways and calcium and ROS homeostasis play crucial regulatory roles during fiber cell initiation and differentiation, and the *Sus* gene encoding sucrose synthase is also involved [8,17,29]. In this study, the Fisher’s exact test was finished to XinWX and XinFLM at three time points during fiber initiation development (-1, 0 and 1 DPA), and transcription factors such as ERFs, MYBs, WRKYs and Zinc-finger proteins were indicative of a positive regulatory effect on early fiber development, indicating important roles in the fiber initiation. Further, in hormone-associated metabolic pathways, GO terms in JA pathway were particularly enriched, especially AOCs, the key enzymes in JA biosynthesis, which suggests that JA is related to progression of fiber initiation.

Additionally, “Lipid transport” and “asparagine biosynthetic process” was also enriched during fiber initiation development. LTPs transport lipids from the endoplasmic reticulum (ER) to the plasma membrane (PM) and the cell exterior, and are known to be associated with fiber elongation [50,51]. LTPs are usually activated during the early stages of fiber development [5], and expression levels peaked at around 10 DPA [52]. Arfinine is the predominant nitrogen storage unit in cotton and accounts for at least 25% of total stored nitrogen, and the majority of nitrogen transported between cotyledons and the growing axis is via the asparagine pathway [53]. Nitrogen availability strongly influences cotton fiber yield and quality, and changes in nitrogen concentration can also influence sucrose inversion and the activity of invertase, sucrose synthetase and sucrose phosphate synthetase [54,55]. In this study, LTPs and genes associated with asparagine biosynthesis were upregulated in the microarray data of XinFLM compared to that of XinWX at 0 and 1 DPA, indicating important roles in the early stages of fiber development for these proteins. Further functional characterization is required to explore this possibility.

### Allene oxide cyclases and Jasmonic acid-associated metabolism regulate JA and affect fiber initiation

In plants, JA is synthesized from α-linolenic acid, which is oxygenated by 13-lipoxygenase. Allene oxide synthase (AOS) and allene oxide cyclase activity subsequently results in cis-(+)-12-oxo-phytodienoic acid (OPDA). AOC is required for formation of the cis-(+)-enantiomer (9S, 13S) of OPDA, which is thought to be the precursor of JA [56]. In *Medicago truncatula*, suppressing *MtAOC1* expression in roots lowered JA levels significantly [37], which highlighted the importance of AOC in JA biosynthesis. Genes encoding enzymes involved in JA biosynthesis generally induced by JA in *Arabidopsis* and tomato, indicating feed-forward regulation [38, 57]. JA, OPDA and their derivatives can induce *AOC* expression in *Arabidopsis* [58]. Biotic or abiotic stresses that lead to endogenous increases in octadecanoids and jasmonates are usually accompanied by upregulation of *AOC*, *AOS* and *OPR3* transcription [59–61]. *AOC* transcription and JA levels therefore appear to be coordinated during plant growth and development.

In this study, *AOC*, which was up-regulated in XinWX, was also enriched in XinFLM at 1 DPA compared to -1 DPA. The expression pattern of *AOCs* in different tissues, organs, and fibers at different development stages in TM-1 showed that they had preferential expression in fiber initiation stage (-3~1 DPA), especially at -1 DPA, suggesting a critical regulatory role in fiber initiation. Additionally, expression was significantly higher in lintless-fuzzless mutants than in linted-fuzzless and linted-fuzzed accessions, especially at -1 DPA, indicating that overexpression of *AOCs* may have a negative effect on progression of fiber initiation in lintless-fuzzless lines. These results, in combination with SEM images that showed no protrusions during fiber cell initiation at the ovular surface at 0 DPA in XinWX, indicated that *AOCs* are important for progression of fiber initiation but may suppress this process upregulated above a threshold value.

The development of cotton fibers shares many similarities with leaf trichome development in *Arabidopsis*, in which exogenous application of JA increases the number and density of leaf trichomes and has a synergistic effect on trichome induction with Gibberellin [62–65]. In cotton, continuous exogenous JA application inhibited fiber initiation and elongation, and this effect was dependent on both dosage and development stage [66]. *GhTCP*, which directly participates in the regulation of JA biosynthesis, influences fiber elongation via a complex system. Overexpression of *GbTCP* in cotton fibers increased the JA content but resulted in fewer fibers that were also shorter. However, in *GbTCP*-silenced cotton lines, the mature fibers were also shorter with lower quality [67], indicating that fiber elongation requires an optimal JA concentration. During fiber initiation, JA content at -1 DPA ovules is also higher, and JA response genes are up-regulated [66]. In this study, “jasmonic acid mediated signaling pathway” was enriched within XinWX suggested JA involved in fiber initiation. JA induced the expression of *AOCs* in cotton, with significant upregulation between 4 and 12h, and a peak in expression at 8 h. The expression profiles showed four *AOCs* and other genes encoding the main JA biosynthetic enzymes were up-regulated, indicating a high level of JA during the fiber initiation stage. However, expression levels were significantly higher in XinWX than in XinFLM, therefore JA appears to be related to normal fiber initiation in cotton, but overproduction of JA may suppress fiber development. Furthermore, we also sampled -2 DPA ovules from TM-1 treated with JA of different concentrations. After 2 days of culture of exogenous JA application, we found as low as 0.1μM JA to inhibit severly fiber initiation, and the inhibition was dose dependent (S2 Fig). This confirmed that high concentration of JA suppressed fiber development.

Cotton fiber initiation is a complex process involving many different plant hormones. *GH3*.*3*, *GH3*.*5*, *GH3*.*6* and *GH3*.*11*, all of which are induced by auxin, have been shown to be associated with JA homeostasis during initiation of adventitious roots in *Arabidopsis* [43]. *ARF6* and *ARF8*, transcription factors that are also transcriptionally regulated by auxin, are positive regulators of JA biosynthesis during flower development [68,69]. MYC2, a bHLH transcription factor, have been reported to play a central role within the JA signaling pathway in regulating diverse JA responses [32]. Furthermore, expression of the plant defense gene *ERF1* is rapidly activated by either ethylene or jasmonate, and synergistically by both hormones [70]. In this study, two GH3 proteins were differentially expressed in XinWX and XinFLM, with higher levels of expression in XinWX. This is consistent with the expression patterns observed for JA biosynthesis-associated genes. In summary, there appeared to be crosstalk between different hormones during fiber development in cotton. The exact regulatory mechanisms of the cross-talk between JA and other hormones require further investigation.

## Conclusions

Developmental mutants are ideal for exploring the key genes and pathways in plant fiber development. In this study, several important genes and pathways involved in cotton fiber initiation were identified, including hormone-associated metabolic pathways, lipid transport and asparagine biosynthetic processes, and various transcription factors. Additionally, JA was found to be related to normal fiber initiation, but overproduction of JA is unfavorable for fiber development.

## Supporting Information

S1 Table Gene-specific primers used for RT-qPCR analysis. (XLS)

S2 Table Complete information on over-represented GO terms within XinWX and XinFLM. (XLS)

S3 Table Complete information on over-represented GO terms between XinWX and XinFLM. (XLS)

S4 Table Statistical significance analysis of the expressions of *AOCs* in fiber mutant lines at -1 DPA. A, B, C, D, E indicated significant differences of expressional level (P<0.01) among different lines. The same alphabet represented no significant difference.
(XLS)

S1 Fig Phenotypic characterization of XinWX and XinFLM. (TIF)

S2 Fig Scanning electron microscopy analysis of the fiber development using -2 DPA ovules in TM-1 after 2 days of culture of exogenous JA application. (1) ovules cultured with standard BT medium; (3) ovules treated with 0.1μM JA; (5) ovules treated with 0.5μM JA; (7) ovules treated with 2.5μM JA. (2), (4), (6), and (8) are the enlarged views of the fiber initiation section in (1), (3), (5), and (7), respectively. The magnification in (1), (3), (5) and (7) is 50×, and in (2), (4), (6) and (8) is 300×. Arrows indicated the protrusion of epidermal cells.
(TIF)
