# Supplementary figures and images for: Comparative Transcriptomics Reveals Jasmonic Acid-Associated Metabolism Related to Cotton Fiber Initiation
Source: PLoS One. 2015 Jun 16;10(6):e0129854. doi: 10.1371/journal.pone.0129854 (PMC4469610; doi:10.1371/journal.pone.0129854)

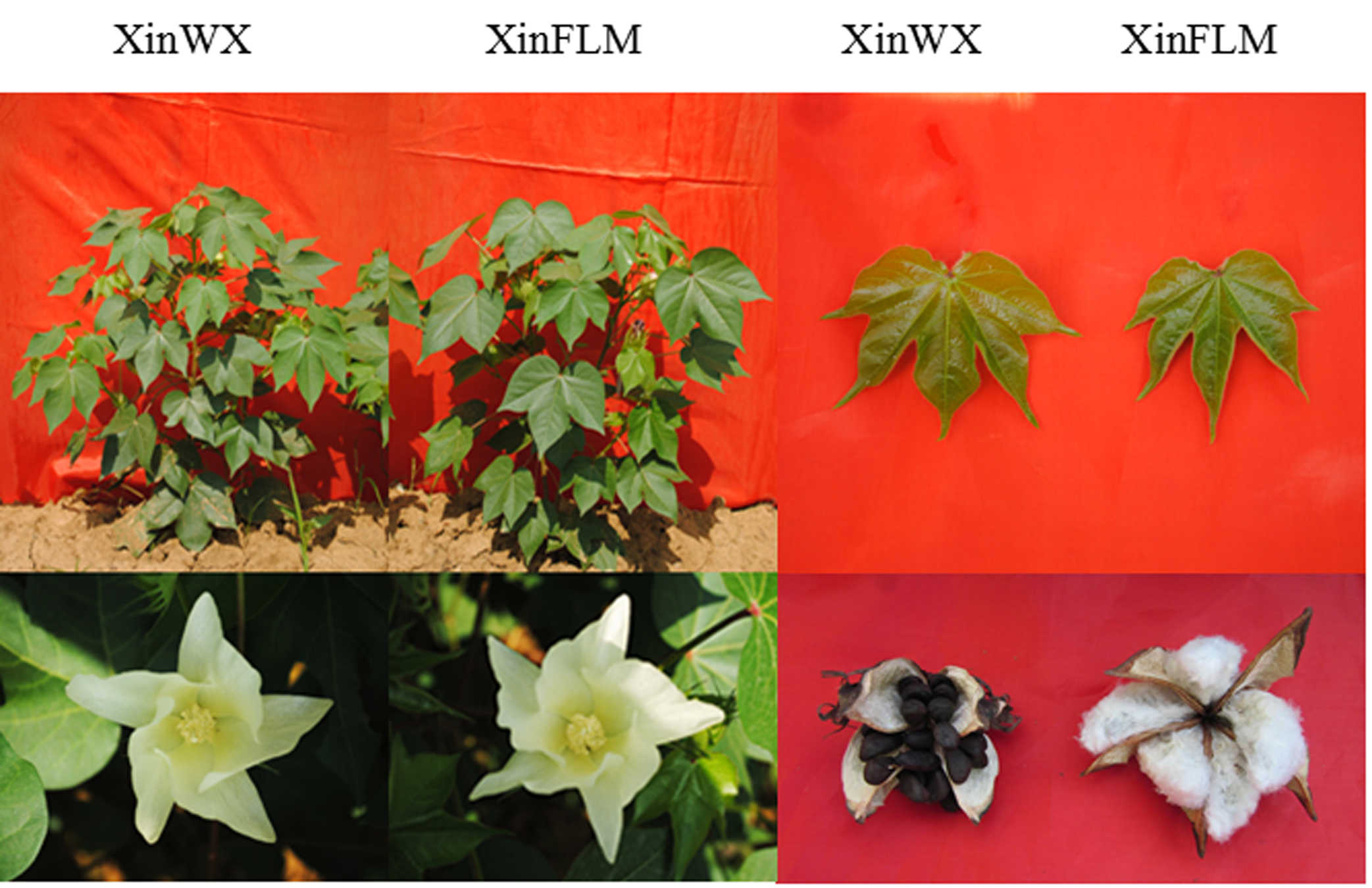

Supplement: S1 Fig — (TIF) [file pone.0129854.s005.tif]

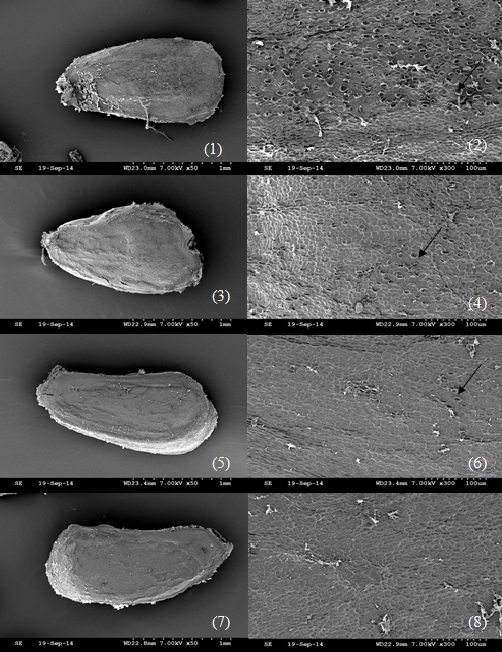

Supplement: S2 Fig — (1) ovules cultured with standard BT medium; (3) ovules treated with 0.1μM JA; (5) ovules treated with 0.5μM JA; (7) ovules treated with 2.5μM JA. (2), (4), (6), and (8) are the enlarged views of the fiber initiation section in (1), (3), (5), and (7), respectively. The magnification in (1), (3), (5) and (7) is 50×, and in (2), (4), (6) and (8) is 300×. Arrows indicated the protrusion of epidermal cells. (TIF) [file pone.0129854.s006.tif]
